# The Role of Dance in Stroke Rehabilitation: A Scoping Review of Functional and Cognitive Effects

**DOI:** 10.3390/jcm14165742

**Published:** 2025-08-14

**Authors:** Roberta Lombardo, Gabriele Triolo, Daniela Ivaldi, Angelo Quartarone, Viviana Lo Buono

**Affiliations:** IRCCS Centro Neurolesi “Bonino-Pulejo”, 98124 Messina, Italy; roberta.lombardo@irccsme.it (R.L.); daniela.ivaldi@irccsme.it (D.I.); angelo.quartarone@irccsme.it (A.Q.); viviana.lobuono@irccsme.it (V.L.B.)

**Keywords:** stroke, rehabilitation, dance, balance, gait, quality of life

## Abstract

**Background:** Stroke remains a leading cause of long-term disability globally, frequently resulting in persistent motor and cognitive impairments. Rehabilitation is critical for promoting recovery, and dance-based interventions have emerged as a promising complementary approach. **Objective:** This scoping review aimed to examine the recent literature on the application of dance in stroke rehabilitation, with a particular focus on its effects on motor function (including balance and gait), cognitive performance, and quality of life. **Methods**: A comprehensive literature search was conducted across four electronic databases, PubMed, Embase, Web of Science, and Scopus, between January and March 2025. Studies were eligible for inclusion if they involved adult participants with a history of stroke, implemented dance-based rehabilitation interventions, and reported outcomes related to motor function, cognition, or psychosocial well-being. The review process adhered to the PRISMA-ScR (Preferred Reporting Items for Systematic Reviews and Meta-Analyses extension for Scoping Reviews) guidelines. **Results:** Of the 778 records initially identified, four studies met the inclusion criteria: two randomized controlled trials, one interventional study, and one feasibility study. Overall, dance-based interventions were associated with improvements in dynamic balance, gait coordination, and general mobility. Furthermore, the interventions demonstrated high levels of adherence and participant satisfaction. **Conclusions**: Dance-based rehabilitation may offer meaningful motor benefits for individuals recovering from stroke, particularly in improving balance, gait, and overall mobility. However, the heterogeneity of intervention protocols and the limited assessment of cognitive and psychosocial outcomes underscore significant gaps in the current literature. To establish the efficacy and underlying mechanisms of dance-based approaches, future research should prioritize high-quality randomized controlled trials employing standardized intervention designs and comprehensive outcome measures, including cognitive and quality-of-life domains.

## 1. Introduction

Stroke is a medical emergency condition involving brain tissue damage or death [1]. Stroke can be broadly categorized into ischemic stroke and hemorrhagic stroke. Ischemic stroke represents 71% of all strokes globally [2] and is caused by the obstruction or reduction of blood flow in a cerebral artery. As recently highlighted in the literature, ischemic stroke is considered a major contributor to severe disability worldwide [3]. In contrast, hemorrhagic stroke can result from hypertension, rupture of an aneurysm or vascular malformation, or as a complication of anticoagulation medications. An intracerebral hemorrhage occurs when bleeding takes place directly within the brain tissue, typically resulting in the formation of a clot [4]. It is caused by the rupture of a blood vessel, which not only leads to neuronal damage but also triggers an inflammatory cascade. This cascade promotes edema and tissue compression, ultimately impairing the function of the affected brain region [5].

Common impairments caused by stroke can include limb weakness, urinary incontinence, dysphagia, impaired consciousness, speech alteration, cognitive impairment, and loss of spatial perception. Motor impairment is the most common stroke-related issue, with upper limb weakness occurring more frequently than lower limb weakness. Specifically, 77% of stroke patients experience upper limb weakness, while 72% experience weakness in the lower limbs [6]. In line with these findings, Dalton et al. [7] identified upper limb coordination, delayed recall, and upper limb light touch sensation as the most common post-stroke impairments among the ten domains assessed.

Rehabilitation plays a crucial role in the continuum of care for stroke patients.

Comprehensive stroke rehabilitation strategies, including physical, occupational, and speech therapy, are crucial for optimizing recovery outcomes [8]. Complementary and alternative non-motor therapies, such as psychosocial interventions or Cognitive Behavioral Therapy, demonstrate promising effects in alleviating post-stroke depression and show potential in reducing post-stroke fatigue [9,10].

Post-stroke rehabilitation has traditionally focused on training patients in compensatory strategies to restore balance, gait, and independence in activities of daily living. Goal-directed exercises involve setting specific, measurable objectives tailored to personal activities, such as dressing independently or walking a defined distance, thereby increasing patient engagement in the recovery process. Research has demonstrated that goal setting significantly enhances stroke rehabilitation outcomes by providing clear targets and promoting patient autonomy [8]. Motor control improvement leverages proprioceptive training, emphasizing body position awareness and coordination to reduce fall risk. Exercise specificity advances through task-specific training, incorporating greater use of both proximal and distal movements during intensive real-world activity practice. The volume of exercise is likely a key determinant of functional gains, depending on the individual’s level of residual motor capacity [11]. Recent research has highlighted new evidence suggesting that accurate prediction of treatment response may enable earlier and more targeted interventions, thereby enhancing motor recovery. Predictive models, particularly those based on the initial severity of motor impairment, can help inform decisions regarding the type, dose, intensity, and duration of the intervention [12].

In this context, dance represents a complex activity, encompassing various forms of expression that are present across cultures worldwide. It typically involves structured, rhythmic movements performed in both spatial and temporal dimensions, accompanied by music, conveying emotions or ideas, or serving as an intrinsic source of enjoyment. Dance can be performed individually, in pairs, or within groups, incorporating either choreographed sequences or spontaneous improvisation. It integrates techniques such as visual focus, rhythmic synchronization, imagery, proprioceptive awareness, and the replication of distinct movement patterns. Furthermore, dance provides an opportunity for training dual-tasking abilities [13].

Dance therapy has been shown to reduce depression and anxiety, enhance quality of life, and improve interpersonal, cognitive, and motor skills. For this reason, dance therapy can serve as a complementary approach to conventional physiotherapy by improving motor function, addressing cognitive deficits, and promoting both the social and physical well-being of people with stroke [14,15,16]. Physiotherapy encompasses a wide range of treatment modalities, and recent studies conducted in individuals with Parkinson’s disease have shown that dance can have beneficial effects on motor symptoms, balance, and gait [17,18].

This review aims to investigate whether dance can serve as a viable alternative or complementary approach to traditional physiotherapy for improving motor and cognitive deficits in post-stroke survivors. While dance has shown promising benefits in other neurological conditions, its application in stroke rehabilitation is still emerging, with the current evidence being limited and methodologically heterogeneous.

This review analyzed a variety of dance styles, including Argentine tango, contemporary dance, folk dance, jazz, and rhythm-based movement, to capture the diversity of approaches used in rehabilitation contexts. Specifically, the review seeks to (1) describe the characteristics of dance-based interventions in post-stroke populations using the FITT principle (Frequency, Intensity, Time/Duration, and Type); and (2) evaluate the available evidence on their effects on key rehabilitation outcomes, such as balance, gait, fall risk, cognitive impairment, and overall feasibility, compared to conventional physiotherapy.

By synthesizing findings across multiple studies, this review aims to provide a clearer understanding of the potential role of dance in stroke rehabilitation and identify directions for future research and clinical practice.

## 2. Materials and Methods

A scoping review was conducted to explore the recent scientific literature on the effects of dance in individuals with stroke, focusing specifically on motor aspects such as balance and gait, cognitive impairment, and the tolerability of this approach. The objective is to affirm the effectiveness of rehabilitation training in the form of dance, aiming to implement it as an alternative approach to traditional rehabilitation methods.

A comprehensive literature search was conducted between January and March 2025 across the databases PubMed, Embase, Web of Science, and Scopus. Studies published up to the time of the search were included, with no restrictions on publication date. Only articles written in English were considered to ensure consistency in data analysis and methodological coherence. However, this language restriction may have introduced a potential bias by excluding relevant studies published in other languages. A combination of keywords was used, including “(((dance) OR (dance rehabilitation) OR (dance therapy) AND (stroke)”. A complete overview of the keywords used in the search strategy is provided in Table 1.

The screening process involved duplicate removal and the application of predefined eligibility criteria. All stages of study selection were independently carried out by the lead reviewers (R.L., G.T., and D.I.), including title and abstract screening as well as full-text assessment. Any disagreements regarding study selection were first discussed among the reviewers and resolved by consensus; if necessary, a fourth investigator (V.L.B.) was consulted to make the final decision. To assess the methodological quality of the included studies, a risk of bias evaluation was performed using the critical appraisal tools developed by the Joanna Briggs Institute (JBI) [19,20,21,22], selected according to the study design (randomized controlled trial or quasi-experimental study). Two authors (R.L. and G.T.) independently assessed each methodological criterion, and the fourth reviewer (L.B.V.) supervised the process and resolved any disagreements regarding study inclusion. The results of the assessment are presented in Table 2 and Table 3.

**Figure 1:**
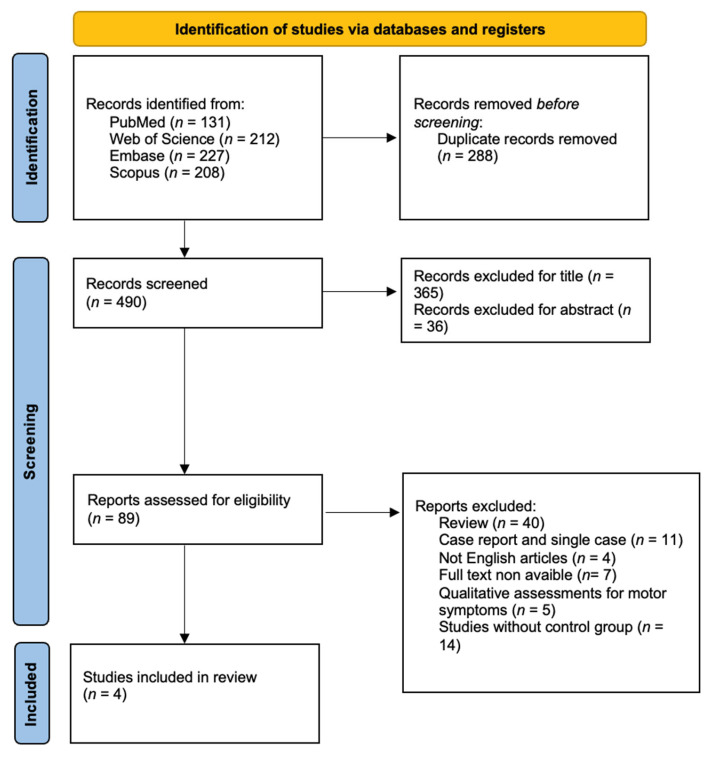
PRISMA flow chart.

The review methodology adhered to the Preferred Reporting Items for Systematic Reviews and Meta-Analyses (PRISMA) framework (Figure 1) [18].

### 2.1. Study Selection

A total of 778 records were identified, of which 288 were excluded as duplicates. Titles and abstracts of the remaining records were screened, resulting in 89 articles undergoing abstract-level review. After full-text screening, 4 studies met the inclusion criteria and were included in the final analysis. The limited number of included studies results from the rigorous exclusion of studies lacking a control group, a criterion applied to ensure the validity of the findings; however, this approach reduces the generalizability of the evidence and the robustness of the conclusions.

### 2.2. Inclusion Criteria

Studies were included if they met the following criteria: (a) studies enrolled adult participants (>18 years old) with a clinical diagnosis of stroke, both ischemic and hemorrhagic; (b) studies implemented a dance-based intervention, either in individual or group settings, administered by physiotherapists or dance professionals; (c) studies comparing dance-based interventions to conventional rehabilitation methods or to no intervention; and (d) studies using standardized scales for motor outcomes.

### 2.3. Exclusion Criteria

Studies that did not meet the following criteria were excluded: (a) studies involving populations with other neurological or non-neurological conditions; (b) systematic reviews or meta-analyses; (c) case reports and single-subject studies; (d) studies published in languages other than English; (e) studies using qualitative evaluation criteria for objective measures; and (f) studies without a control group.

## 3. Results

We found 778 studies; once the duplicates were eliminated, we analyzed 490 studies. The remaining articles were screened in relation to title, abstract, and in relation to inclusion and exclusion criteria. Finally, four articles were validated for analysis: one feasibility study [22], one non-randomized controlled study [21], and two randomized controlled trial studies [19,20].

Studies evaluate the effectiveness of dance as an alternative intervention to conventional therapy or as a novel therapeutic approach for people with stroke.

All included studies examined motor improvements attributable to dance-based rehabilitation training; of these, only three also assessed intervention feasibility, one study incorporated a measure of participant satisfaction, and a single study within the established inclusion criteria reported concomitant gains in cognitive domains (see Table 4).

To highlight the key findings, Table 5 presents a synthesis of the major improvements reported in the selected studies.

### 3.1. Gait and Balance

Several studies have examined the impact of dance interventions on balance, functional mobility, and gait. Patterson et al. [22] found no significant differences in the spatiotemporal parameters of gait; however, participants demonstrated notable improvements in balance, particularly during dynamic gait.

In contrast, Bruyneel et al. [19] reported that after 4 and 6 weeks of dance intervention, Mini-BESTest scores significantly increased for the dance group, whereas the conventional treatment group showed no significant changes at either follow-up. At the final follow-up, participants in the dance group had a higher Mini-BESTest score compared to the conventional treatment group, though this difference did not reach statistical significance. However, relative to balance confidence, no differences were found between the groups.

In the study by Varga et al. [21], significant improvement was found in the dance group for the Dynamic Gait Index and Five Times Sit to Stand Test. These findings suggest that folk music and folk dance, by incorporating dynamic weight-shifting components, enhance dynamic postural control and balance. Therefore, the results support existing evidence that chronic stroke subjects may benefit from additional folk dance sessions, particularly in improving dynamic balance components.

Young et al. [20] also reported findings like those of other studies. When comparing the 6-Minute Walking Test and Five Times Sit to Stand Test parameters to baseline, although no statistically significant differences were found, the group receiving the M2M treatment demonstrated greater distance and reduced time compared to baseline. This indicated a moderate effect size for improvements in lower limb endurance and functional strength. No significant differences were reported between the groups regarding the Timed Up and Go Test after the intervention.

### 3.2. Cognitive Impairments and Quality of Life

The only study that reported data on cognitive impairment and quality of life was conducted by Bruyneel et al. [19], who used the Montreal Cognitive Assessment to assess cognitive deficits and the Stroke Specific Quality Of Life scale to evaluate quality of life. However, no significant differences were found between testing sessions or between groups.

### 3.3. Satisfaction

Bruyneel et al. [19], using the Dance Satisfaction Questionnaire, found that the most commonly reported benefits of dance practice were the enjoyment of the activity and improvements in balance and coordination. Additionally, the majority of participants expressed a desire to continue dancing after being discharged from the rehabilitation center.

### 3.4. Narrative Summary of Outcome Consistency and Variability

Despite differences in study design, sample size, intervention protocols, and outcome measures, the overall results of the included studies show a moderate degree of consistency, particularly regarding motor improvements. All four studies reported improvements in balance, gait, or functional mobility, with the most significant improvements observed in dynamic balance and lower limb strength [19,20,21,22]. In contrast, results related to cognitive and psychosocial outcomes were heterogeneous and limited, with only one study assessing cognitive performance and quality of life [19], which did not produce statistically significant changes. In addition, satisfaction was reported in two studies [19,22] using self-assessment tools, indicating high acceptability but lacking standardized quantification. Therefore, while motor outcomes show promising and relatively consistent improvements, cognitive and psychosocial outcomes remain inconclusive due to variability in measurement and limited data availability.

## 4. Discussion

This review aimed to evaluate the effectiveness of dance as a rehabilitation strategy for post-stroke subjects, with a focus on motor outcomes, cognitive functioning, and quality of life. Current evidence indicates that dance-based rehabilitation intervention yields significant improvements in motor functioning [19,21,22], such as dynamic balance, motor coordination, and general functional abilities [19,20]. Micheli Rochetti et al. [23] in a pilot study assessing the effects of Bolero, a Cuban dance similar to tango, reported improvements in all measures of balance and functional mobility (Berg Balance Scale, Functional Reach Test, Timed Up and Go Test).

Bognar et al. [24] reported perceived improvements among stroke patients after dance intervention, particularly regarding body posture adjustments and maintenance (e.g., lying down, sitting, standing up), as well as fine motor skills involving hand movements. Benefits in joint mobility, muscle strength, and tone were noted, subsequently impacting balance and voluntary movement coordination. This facilitated moderate improvements in walking.

When employed as an alternative or adjunct to conventional rehabilitation, dance has also been associated with enhanced self-perception and improved quality of life in stroke survivors [19]. These benefits may arise not only from physical gains, but also from the enjoyment and engagement fostered by the activity. Rhythmic and coordinated movement patterns inherent to dance are believed to stimulate neural plasticity and promote recovery of motor and non-motor domains [21]. In addition, dance programs have demonstrated psychosocial benefits such as reduced stress, increased emotional well-being, and improved social connectedness [22,24].

Dance training stimulates neural plasticity through rhythmic and coordinated movements, positively affecting motor but also cognitive and emotional functions in stroke survivors [21].

Dance-based rehabilitation also provided notable social and stress-reduction benefits.

The most frequently reported positive effects were related to enhanced mood and motivation, with dance activities fostering greater engagement in the rehabilitation process and boosting participants’ confidence in their abilities. Many participants indicated that attending dance sessions lifted their spirits and offered an enjoyable and meaningful activity to look forward to. They reported feeling more relaxed, energized, and emotionally connected during and after the sessions [24]. Several factors contributed to this emotional benefit, including social interaction with classmates and the energy generated during dancing. The choice of music and the instructor’s approach were also reported as having a strong influence on participants’ emotional states. Although occasional negative emotions such as frustration due to physical limitations or embarrassment from being observed were mentioned, participants emphasized that witnessing others’ progress and sharing enjoyable moments enhanced their own experience and increased the perceived value of the class [24].

Macko [25] highlighted the positive impact of mobility-focused exercises on depression scores.

In group settings in particular, dance appeared to mitigate depressive symptoms and apathy [26,27,28,29,30] and improved cognitive domains such as flexibility, processing speed, and memory.

Bruyneel et al. [19] suggest that the frequency of rehabilitation sessions may be a critical factor in achieving meaningful improvements in cognition and quality of life. Together, they emphasize the importance of both the type of intervention and its frequency in determining rehabilitation outcomes. As reported by Bognar et al. [24], dance-based intervention promoted social integration, noting that stroke subjects initially unwilling to engage socially later formed meaningful relationships through dance sessions, expressing a desire to continue even after the project’s completion. Also, in a hospital-based rehabilitation setting, rehabilitation training using dance improved to enhance social interactions, progress awareness, and feelings of accomplishment [31].

Collectively, dance interventions are feasible across different recovery phases and settings, with perceived psychosocial benefits potentially supporting broader rehabilitation outcomes.

In addition, the available evidence consistently indicates a high degree of feasibility for dance-based interventions. The studies analyzed indeed reported significant adherence rates, high levels of participant satisfaction, and minimal adverse events, supporting the overall practicality and acceptability of these programs [21,22]. Such feasibility is particularly relevant as it can foster patient engagement and long-term adherence, both crucial factors for successful rehabilitation outcomes.

This scoping review is subject to several limitations. First, the number of eligible studies was small, limiting the generalizability of findings. Second, the included studies displayed substantial methodological heterogeneity in terms of dance style, intervention intensity, frequency, duration, and outcome measures. The heterogeneity of interventions across studies compromises comparability and limits the identification of evidence-based, standardized parameters. Sample sizes were generally small, reducing statistical power and increasing the risk of error. Additionally, while motor outcomes have been widely assessed, cognitive effects have rarely been explored and reported, leaving a critical knowledge gap. Additional potential sources of bias warrant consideration. None of the studies has systematically accounted for confounding variables, including instructor-related factors (e.g., differences in training background, communication style, or rehabilitation experience), music selection, or the characteristics of the activity setting. These elements may significantly influence participants’ motivation and engagement. The absence of standardized training protocols and long-term follow-up further constrains the interpretation and replicability of results, contributing to the heterogeneity of observed outcomes. Finally, the predominance of feasibility and early-phase studies highlights the need for well-powered randomized controlled trials with robust methodology to confirm and extend current evidence. Current evidence on the use of specific dance styles in post-stroke rehabilitation is limited, making it difficult to determine the most effective intervention. Additionally, detailed information regarding intervention delivery and music selection is scarce. Given that each dance genre involves unique stylistic features, cognitive demands, and musical stimuli, outcomes may vary depending on the chosen style [23]. Some interventions incorporated complex and dynamic, predominantly standing movements requiring substantial physical engagement, whereas others involved seated exercise and simpler, lower-intensity aerobic components. Such variability in motor load and cognitive stimulation may have influenced the effectiveness of interventions on specific outcomes, such as balance, lower limb strength, or fatigue. Only a few protocol studies have been identified outlining large-scale randomized controlled trials designed to investigate the effect of adapted dance forms on balance, functional mobility, strength, coordination, cognitive functions, motivation, and quality of life in stroke survivors [32,33,34]. Moreover, group dance interventions have been associated with improvements in motor function, quality of life, and cognitive performance in individuals with Parkinson’s disease [27,28,29,30,35]. Similarly, in populations with mild cognitive impairment, Alzheimer’s disease, or dementia, dance has shown benefits in maintaining cognitive function and reducing depressive symptoms [26,27,28,36]. However, despite growing interest and supportive evidence in these neurological conditions, relatively few studies have specifically investigated the impact of dance as a rehabilitation approach for stroke survivors. To address these critical issues, future studies should adopt standardized, replicable, and well-documented protocols, paying particular attention to interpersonal and environmental factors that may influence outcomes. Moreover, interventions should include clearly defined parameters—such as session frequency, duration, intensity, and type of dance—to enhance comparability across studies. Researchers are also encouraged to incorporate appropriate control groups and follow-up assessments to evaluate the sustainability of outcomes over time.

In light of current evidence and the gaps identified, future studies should adopt interventions structured according to the FITT principle, in order to more precisely define the optimal parameters for post-stroke rehabilitation through dance and enable the development of standardized protocols. Regarding frequency, preliminary findings suggest that at least 2–3 sessions per week, delivered over a minimum period of 6 to 12 weeks, may be necessary to achieve significant improvements in balance and functional mobility.

As for intensity, the use of standardized subjective scales such as the Borg Rating of Perceived Exertion (RPE), targeting values between 11 and 14 (“light” to “somewhat hard”), is recommended to ensure an appropriate yet safe cardiovascular load for individuals with stroke sequelae. The duration of sessions should also be harmonized: interventions lasting 45 to 60 min, structured into warm-up, choreographed core activity, and cool-down phases, appear appropriate.

Concerning dance style, future research should systematically compare structured styles (e.g., folk dance, adapted tango) with more free-form approaches (e.g., improvisational or creative dance), to evaluate differences in motor, cognitive, and emotional impact. Finally, the inclusion of subgroup analyses represents a highly justified direction for future research. Response to intervention may vary according to clinical and demographic variables such as age, sex, time since the acute event, and degree of residual disability. Such analyses would allow for the identification of specific subgroups of patients who may derive the greatest benefit from tailored dance-based rehabilitation protocols.

## 5. Conclusions

Dance-based interventions consistently demonstrated improvements in dynamic balance, gait coordination, and overall functional mobility in stroke survivors. Moreover, they have been associated with high adherence rates, participant satisfaction, and minimal adverse events, emphasizing their acceptability and practicality.

Future studies should prioritize the implementation of robust randomized controlled trials directly comparing dance-based rehabilitation with conventional physiotherapy to determine the true efficacy of this approach. Standardized training protocols must also be developed, with clear specifications regarding session frequency, duration, intensity, and dance style, music, setting, and instructor characteristics. Furthermore, future research should specifically explore the mechanisms by which dance facilitates motor and cognitive recovery and how these benefits contribute to improved quality of life and social integration for stroke survivors. Addressing these gaps will support the broader integration of dance into comprehensive stroke rehabilitation programs.

## Figures and Tables

**Table 1:** Derived keywords used for generation of the initial data set.

"(((dance) OR (dance rehabilitation)) OR (dance therapy)) AND (stroke)" ("danced"[All Fields] OR "dancing"[MeSH Terms] OR "dancing"[All Fields] OR "dance"[All Fields] OR "dances"[All Fields] OR (("danced"[All Fields] OR "dancing"[MeSH Terms] OR "dancing"[All Fields] OR "dance"[All Fields] OR "dances"[All Fields]) AND ("rehabilitant"[All Fields] OR "rehabilitants"[All Fields] OR "rehabilitate"[All Fields] OR "rehabilitated"[All Fields] OR "rehabilitates"[All Fields] OR "rehabilitating"[All Fields] OR "rehabilitation"[MeSH Terms] OR "rehabilitation"[All Fields] OR "rehabilitations"[All Fields] OR "rehabilitative"[All Fields] OR "rehabilitation"[MeSH Subheading] OR "rehabilitation s"[All Fields] OR "rehabilitational"[All Fields] OR "rehabilitator"[All Fields] OR "rehabilitators"[All Fields])) OR ("dance therapy"[MeSH Terms] OR ("dance"[All Fields] AND "therapy"[All Fields]) OR "dance therapy"[All Fields])) AND ("stroke"[MeSH Terms] OR "stroke"[All Fields] OR "strokes"[All Fields] OR "stroke s"[All Fields]).

**Table 2:** Critical appraisal of eligible quasi-experimental.

Citation	Q1	Q2	Q3	Q4	Q5	Q6	Q7	Q8	Q9
Patterson, K. K. et al. (2018) [22].	Y	N/A	Y	Y	N	Y	Y	Y	Y
Varga, L. et al. (2023) [21].	Y	Y	Y	Y	N	Y	Y	Y	Y
Yes %	100.0	50.0	100.0	100.0	0.0	100.0	100.0	100.0	100.0

**Table 3:** Critical appraisal of eligible randomized controlled trial.

Citation	Q1	Q2	Q3	Q4	Q5	Q6	Q7	Q8	Q9	Q10	Q11	Q12	Q13
Bruyneela, A. V. et al. (2023) [19].	Y	U	N	N	N	Y	Y	Y	Y	Y	Y	Y	Y
Young, H.J. et al. (2021) [20].	Y	U	Y	N	N	Y	Y	Y	Y	Y	Y	Y	Y
Yes %	100.0	0.0	50.0	0.0	0.0	100.0	100.0	100.0	100.0	100.0	100.0	100.0	100.0

**Table 4:** Overview of included studies.

Authors	Study	Study Design	Population	Groups	Adverse Events	Type of Dance/Activity	Frequency of Intervence	Measurerment
Patterson, K. K. et al. (2018) [22]	A dance program to improve gait and balance in individuals with chronic stroke: a feasibility study.	Feasibility study.	22 people with chronic stroke. Two dropped out.	Two groups of 10 participants.	Postoperative data is missing as one patient reported ankle swelling.	The dance program included a variety of choreographed sequences based on ballet, contemporary, jazz, folk, and ballroom styles.	2 sessions/week, 60 min each, for 10 weeks.	NIHSS, CMSA, MoCA, self-report questionnaire.
Bruyneela, A. V. et al. (2023) [19]	Dance after stroke improves motor recovery in the subacute phase: A randomized controlled trial.	A randomized controlled trial.	19 people with subacute stroke (within the last three months). Three individuals dropped out.	Two groups.	No adverse events reported.	Each session included a warm-up, technical exercises, improvisation, and a short choreographed dance sequence.	Standard rehab 6 weeks (both groups) + 1 dance session/week for 6 weeks (dance group only).	Mini-BESTest, FIM, ABC scale, LEMOCOT.
Varga, L. et al. (2023) [21]	The Benefits of Enhanced Folk Dance Sessions for Stroke Rehabilitation in Terms of Motor Learning.	Non-randomized controlled study.	24 people with chronic stroke.	Two groups.	No adverse events reported.	Experimental group: conventional physical therapy plus additional folk dance sessions. Control group: conventional physical therapy only.	5 sessions/week, 50 min each, for 3 weeks.	BBS, FSST, TUG, DGI, VAMS, BBT.
Young, H.J. et al. (2021) [20]	The Effects of a Movement-to-Music (M2M) Intervention on Physical and Psychosocial Poststroke: A Randomized Controlled Outcomes in People Poststroke: A Randomized Controlled Trial.	Randomized controlled trial.	People with chronic stroke (6 months post-stroke).	Two groups.	Three minor adverse events were reported: one fall during a supported movement, one case of pre-session fatigue with hypotension, and one instance of transient post-session tachycardia.	Experimental group: sessions included seated warm-up, upper/lower extremity strengthening, cardio, static and dynamic balance exercises, and cool-down, all choreographed to music. Control group: received biweekly educational newsletters by mail.	3 sessions/week, 60 min each, for 12 weeks.	6MWT, 5TSTS, TUG, self-reported fatigue and pain interference (past 7 days).

Legend: NIHSS = National Institutes of Health Stroke Scale; CMSA = Chedoke–McMaster Stroke Assessment; MoCA = Montreal Cognitive Assessment; Mini-BESTest = Mini Balance Evaluation System Test; FIM = Functional Independence Measure; ABC scale = Activities-Specific Balance Confidence scale; LEMOCOT = Lower Extremity Motor Coordination Test; BBS = Berg Balance Scale; FSST = Four Square Step Test; TUG = Timed Up and Go Test; DGI = Dynamic Gait Index; VAMS = Visual Analog Mood Scales; BBT = Box and Blocks; 6MWT = 6-Minute Walking Test; 5TSTS = Five Times Sit to Stand Test.

**Table 5:** Overview of study outcomes.

Author (Year)	Major Motor Improvement	Other Improvement
Patterson, K.K. et al. (2018) [22]	A limited improvement was observed in gait and balance performance.	Participants reported a high level of satisfaction with the program.
Bruyneela, A.V. et al. (2023) [19]	Significant improvement in Mini-BESTest scores in the dance group (*p* ≤ 0.022).	Secondary outcomes were also positive, indicating overall beneficial effects of the intervention.
Varga, L. et al. (2023) [21]	Both groups showed significant improvements in most assessed parameters; the dance group showed significant improvements in dynamic gait and balance in contrast to static balance.	Significant mood improvement observed only in the dance group (*p* < 0.001), with no significant change in the control group (*p* = 0.091).
Young, H.J. et al. (2021) [20]	M2M participants showed a mean increase of 37.9 m in 6MWT distance, a 6.1 s reduction in 5TSST time, and a 6.5-point decrease in fatigue. No significant differences were found for TUG.	Not reported.

Legend: Mini-BESTest = Mini Balance Evaluation System Test; TUG = Timed Up and Go Test; 6MWT = 6-Minute Walking Test; 5TSTS = Five Times Sit to Stand Test.

## Data Availability

Not applicable.
